# Ion Channels as a Potential Target in Pharmaceutical Designs

**DOI:** 10.3390/ijms24076484

**Published:** 2023-03-30

**Authors:** Sheng-Nan Wu

**Affiliations:** 1 Department of Physiology, National Cheng Kung University Medical College, Tainan 70101, Taiwan; snwu@mail.ncku.edu.tw; Tel: +886-6-2353535-5334; Fax: +886-6-2367280; 2 School of Medicine, National Sun Yat-sen University College of Medicine, Kaohsiung 80424, Taiwan

Voltage-gated ion channels are integral membrane proteins that respond to changes in membrane potential with rapid variations in membrane permeability to ions. These ionic channels modulate various important aspects of membrane excitability, which include resting membrane potential, action potential waveform, firing patterns, neurotransmitter release, and postsynaptic potential. Furthermore, their properties are often altered by cellular responses to neurotransmitters and neuropeptides, making Na^+^, Ca^2+^, or K^+^ channels important mediators of long-term effects on synaptic transmission. This section primarily focuses on transmembrane ion channels as the repurposed targets of small molecules. Our priority will be to provide the researchers of this important and exciting field with glimpses into a number of interesting areas that are worthy of further investigation.

The Na^+^Ca^2+^ (NCX) exchanger is recognized as an important regulator of intracellular Ca^2+^ concentration, and it is expressed not only in cardiac sarcolemma but also in the brain, skeletal muscle, and endocrine tissues. KN-R7943 (2-[2-[4-(4-nitrobenzyloxy)phenyl]ethyl]isothiourea) is an isothiourea derivative that is viewed to selectively suppress the reverse mode of NCX isoform 1 with an IC_50_ value of 1.2–2.4 μM. However, a recent study by Wu and Yu [1] provided evidence that disclosed how, in pituitary GH_3_ lactotrophs, cell exposure to KB-R7943 could inhibit a voltage-gated Na^+^ current (*I*_Na_) in concentration-, time- and voltage-dependent manners. The yielded IC_50_ values needed for KB-R7943 suppressed the amplitude of transient *I*_Na_ (*I*_Na(T)_), and late *I*_Na_ (*I*_Na(L)_) were distinguishable (i.e., 11 and 0.9 μM, respectively). Either benzamil or amiloride, also known to be inhibitors of the NCX exchanging current, was reported to inhibit an *I*_Na(L)_ amplitude. When GH_3_ cells were exposed to Ca^2+^-free, Tyrode’s solution, the deactivating *I*_Na_ was progressively decreased in an exponential fashion as the duration of the depolarizing voltage step became prolonged [1].

Deltamethrin (DLT, decamethrin) is a cyclopropanecarboxylate ester obtained by formal condensation between 3-(2,2-dibromvinyl)-2,2-dimethylcyclopanecarboxylic acid and cyano(3-phenoxyphenyl)methanol. Pyrethroids such as DLT and tefluthrin have been shown to modify the gating properties of voltage-gated Na^+^ (Na_V_) channels. In a recent paper by Lin et al. [2], the investigators reappraised whether and how DLT and other related compounds could modify the magnitude and gating of *I*_Na_ in GH_3_ cells. DLT exposure produced a differential and dose-dependent stimulation of *I*_Na(T)_ and *I*_Na(L)_; consequently, the EC_50_ value needed for DL-stimulated *I*_Na(T)_ or *I*_Na(L)_ was estimated to be 11.2 or 2.5 μM, respectively [2]. The *I*_Na(L)_ augmented by DLT was attenuated through the subsequent addition of dapagliflozin or amiloride but not by chlorotoxin. Moreover, during rapid the depolarizing pulse with a rate of 40 Hz, the *I*_Na(L)_ amplitude for each depolarizing pulse and tail *I*_Na_ following the end of high-frequency repetitive stimuli was progressively increased by adding DLT. The recovery time constant following pulse train stimulation with a continued presence of DLT was drastically increased; however, it could be shortened by the further addition of dapagliflozin [2]. Collectively, the modifications by DLT and tefluthrin on the magnitude, gating kinetics, frequency dependence, and hysteretic strength of *I*_Na_ in electrically excitable cells could be different [2].

Lacosamide (LCS), a functionalized amino acid, is an antiepileptic and analgesic drug that is available orally and intravenously in clinical practice. It is administrated in patients with epilepsy and occasionally in patients with trigeminal neuralgia or other neuropathic pain. However, because of the multiple therapeutic effects of this drug, additional electrophysiological actions need to be further explored. A recent report by Wu et al. [3] demonstrated that *I*_Na(T)_ and *I*_Na(L)_ were differentially suppressed with IC_50_ values of 112 and 26 μM, respectively. The hysteretic strength of persistent *I*_Na_ in response to a triangular ramp pulse was inhibited by LCS exposure. The decaying time constant of *I*_Na(T)_ or *I*_Na(L)_ during a train of depolarizing stimuli was shortened through the addition of LCS. Moreover, the fast and slow time constants of recovery from the *I*_Na_ block by the conditioning pulse-train depolarization were increased. Therefore, the results disclosed that LCS could interact with voltage-gated sodium (Na_V_) channels to alter the magnitude, gating, frequency dependence, and voltage-dependent hysteresis of *I*_Na_ in excitable cells [3].

Picaridin (icaridin, 1-piperidinecarboxylic acid 2-(2-hydroxyethyl)-1-methylpropylester) is a cyclic amine and a member of the piperidine chemical family, has been viewed as a synthetic, and is a broad-spectrum arthropod repellent. The repellent and deterrent activities of picaridine have been thought to involve olfactory sensing in mosquitoes and ticks via their interactions with odorant receptor proteins residing in neurons. However, of note, an interesting paper by Shiau et al. [4] reported that picaridine exposure could exert stimulatory action on *I*_Na(T)_ and *I*_Na(L)_ residing in GH_3_ cells. The *I*_Na(L),_ in response to brief step depolarization, was stimulated to a greater extent than *I*_Na(T)_. As a result, effective EC_50_ values needed for picaridin-stimulated *I*_Na(T)_ and *I*_Na(L)_ were yielded as 32.7 and 2.8 μM, respectively [4]. The overall current versus voltage relationship of *I*_Na(T)_ was noticed through a shift in hyperpolarized potential by approximately 20 mV with picaridin exposure, while the quasi-steady-state inactivation curve of *I*_Na(T)_ was shifted to a more depolarized potential of about 5 mV, with no change in the gating charge of the current. As cells were continually exposed to picaridin, the hysteretic magnitude in response to the upright isosceles-triangular ramp voltage became strikingly augmented. As such, the activities inherent in excitable cells can confer susceptibility to perturbations by picaridin or other structurally similar compounds [4].

Carbamazepine (CBZ, Tegretol^®^, 5H-dibenzo[b,f]azepine-5-carboxamide) is an aromatic anticonvulsant that has been used for the treatment of epileptic disorders and neuropathic pain and, specifically, trigeminal neuralgia. This drug has been also been shown to be an adjunctive treatment in schizophrenia or myotonia and as a second-line agent in bipolar disorders. A study by Wu et al. [5] demonstrated that CBZ exposure was able to exert a depressant action on *I*_Na(T)_ and *I*_Na(L)_ in Neuro-2a cells in a concentration-dependent manner. The *I*_Na(L),_ in response to short-step depolarization, was suppressed to a greater extent than *I*_Na(T)_; therefore, the IC_50_ values needed for the suppression of *I*_Na(T)_ and *I*_NaL)_ seen in these cells were 56 and 18 μM, respectively [5]. The recovery of *I*_Na(T)_ inactivation emerged during varying interpulse intervals and slowed in the presence of CBZ; however, the cumulative inhibition of *I*_Na(T)_ evoked by pulse train stimulation was enhanced by adding this drug. The CBZ presence also produced a mild inhibitory effect on the *erg*-mediated K^+^ current in Neuro-2a cells. The differential inhibition by CBZ of *I*_Na(T)_ and *I*_Na(L)_ is of particular significance and may participate in the regulation of electrical behaviors of excitable cells occurring in vivo.

Lutein (xanthophyll, β,ε-carotene-3,3′-diol), derived from a hydride of a (6′R)-β,ε-carotene, is one of the few xanthophyll carotenoids that is believed to exist not only in vegetables and fruits but also in high concentrations that are present in the macula of the human retina. Evidence from human studies suggests that the dietary intake of lutein can lead to the accumulation of lutein in retinal neural tissue, thereby presumably promoting eye and brain health. Alternatively, the beneficial bioactive effects of lutein have been recognized due to its antioxidant and anti-inflammatory properties. However, an important study by Chuang et al. [6] demonstrated the ability of lutein to suppress the hyperpolarization-activated cation current (*I*_h_) with an IC_50_ of 4.1 μM observed in GH_3_ cells. A hyperpolarizing shift of the steady-state activation curve of *I*_h_ was detected in lutein exposure. The hysteretic strength of *I*_h_ activated by a double ramp pulse was also suppressed by adding this compound. Under current-clamp conditions, exposure to lutein can decrease the magnitude of sag potential [6]. Taken together, these data can be interpreted to mean that, besides its antioxidative or anti-inflammatory properties, the presence of lutein is able to suppress the amplitude as well as alter gating and hysteretic behavior of *I*_h_; that the actions of lutein shown in this report would engage in modifications on spontaneous action potentials in different excitable cells.

QO-58 (5-(2,6-dichloro-5-fluoropyridin-3-yl)-3-phenyl-2-(trifluoromethyl)-1*H*-pyrazolol[1,5-a]pyrimidin-7-one) has been demonstrated to be an opener of KCNQx (K_V_7x) channels. It has also been reported to increase the pain threshold of neuropathic pain in a rat model. The ameliorating effect of this compound has been viewed to be closely linked to its activation of KCNQ (K_V_7) channels. However, a paper by Wu et al. [7] demonstrated a dual activation of M-type K^+^ (*I*_K(M)_) and Ca^2+^-activated K^+^ currents (*I*_K(Ca)_) in GH_3_ cells. The presence of QO-58 concentration dependently increased the amplitude of *I*_K(M)_ and *I*_K(Ca)_ with the EC_50_ values of 3.1 and 4.2 μM, respectively. Under cell exposure to QO-58, the steady-state activation curve of *I*_K(M)_ was shifted along the voltage axis to a hyperpolarized potential with no change in the gating charge of the curve. The hysteretic strength of *I*_K(M)_ activated by a triangular ramp pulse was increased by adding QO-58. Moreover, the QO-58 exposure enhanced the opening probabilities of large-conductance Ca^2+^-activated K^+^ channels as well as shifts in the activation curve of the channel toward a less depolarized potential with no change in the single channel conductance of the channel [7]. Collectively, the interaction of QO-58 with M-type K^+^ or large-conductance Ca^2+^-activated K^+^ channels to simulate *I*_K(M)_ or *I*_K(Ca)_ in excitable cells is expected to occur in a concentration and voltage-dependent manner, assuming that similar in vivo findings occur.

Recent investigations have revealed that, through efficient data acquisition with digital-to-analog conversion, the voltage-clamp protocol with varying waveforms (e.g., triangular ramp voltage) can be specifically designed and exploited. As the whole-cell configuration was firmly established, the voltage protocol can be thereafter applied to the tested cells. As a result, the non-linear relationship of different ionic currents versus membrane potential (i.e., voltage-dependent hysteresis) can be activated [8]. As shown in this paper [8], experimental observations have disclosed that several voltage-gated ion channels were noticed as undergoing non-linear hysteresis, which was elicited during triangular ramp voltage. Such voltage-dependent hysteresis is thought to be linked to the voltage-sensing domain of the channel specified. A variety of small molecules that are known to modify the magnitude and gating properties of ionic currents (i.e., *I*_h_, *I*_K(M)_, and *I*_Na(P)_) exerted by several small-molecule modulators are capable of potentially influencing the functional activities of different excitable cells, presuming that in vivo findings occurred.
